# Individualised Positive End-Expiratory Pressure Settings Reduce the Incidence of Postoperative Pulmonary Complications: A Systematic Review and Meta-Analysis

**DOI:** 10.3390/jcm13226776

**Published:** 2024-11-11

**Authors:** Csenge Szigetváry, Gergő V. Szabó, Fanni Dembrovszky, Klementina Ocskay, Marie A. Engh, Caner Turan, László Szabó, Anna Walter, Fadl Kobeissi, Tamás Terebessy, Péter Hegyi, Zoltán Ruszkai, Zsolt Molnár

**Affiliations:** 1 Department of Anesthesiology and Intensive Therapy, Semmelweis University, 1085 Budapest, Hungary; szigetvary.csenge@semmelweis.hu (C.S.);; 2 Centre for Translational Medicine, Semmelweis University, 1085 Budapest, Hungaryfkobeissi30@gmail.com (F.K.);; 3 Emergency Department, Szent György University Teaching Hospital of Fejér County, 8000 Székesfehérvár, Hungary; 4 Hungary National Ambulance Service, 1055 Budapest, Hungary; 5 Hungarian Air Ambulance Nonprofit Ltd., 2040 Budaörs, Hungary; 6 Institute for Translational Medicine, Medical School, University of Pécs, 7623 Pécs, Hungary; 7 Department of Orthopaedics, Semmelweis University, 1085 Budapest, Hungary; 8 Institute of Pancreatic Diseases, Semmelweis University, 1085 Budapest, Hungary; 9 Department of Anaesthesiology and Intensive Therapy, Pest County Flór Ferenc Hospital, 2143 Kistarcsa, Hungary; 10 Department of Anesthesiology and Intensive Therapy, Poznan University of Medical Sciences, 60-806 Poznan, Poland

**Keywords:** PEEP titration, individualised PEEP, postoperative pulmonary complications, respiratory mechanics, intraoperative oxygenation

## Abstract

**Background:** Progressive atelectasis regularly occurs during general anaesthesia; hence, positive end-expiratory pressure (PEEP) is often applied. Individualised PEEP titration may reduce the incidence of postoperative pulmonary complications (PPCs) and improve oxygenation as compared to fixed PEEP settings; however, evidence is lacking. **Methods:** This systematic review and meta-analysis was registered on PROSPERO (CRD42021282228). A systematic search in four databases (MEDLINE Via PubMed, EMBASE, CENTRAL, and Web of Science) was performed on 14 October 2021 and updated on 26 April 2024. We searched for randomised controlled trials comparing the effects of individually titrated versus fixed PEEP strategies during abdominal surgeries. The primary endpoint was the incidence of PPCs. The secondary endpoints included the PaO_2_/FiO_2_ at the end of surgery, individually set PEEP value, vasopressor requirements, and respiratory mechanics. **Results:** We identified 30 trials (2602 patients). The incidence of PPCs was significantly lower among patients in the individualised group (RR = 0.70, CI: 0.58–0.84). A significantly higher PaO_2_/FiO_2_ ratio was found in the individualised group as compared to controls at the end of the surgery (MD = 55.99 mmHg, 95% CI: 31.78–80.21). Individual PEEP was significantly higher as compared to conventional settings (MD = 6.27 cm H_2_O, CI: 4.30–8.23). Fewer patients in the control group needed vasopressor support; however, this result was non-significant. Lung-function-related outcomes showed better respiratory mechanics in the individualised group (Cstat: MD = 11.92 cm H_2_O 95% CI: 6.40–17.45). **Conclusions:** Our results show that individually titrated PEEP results in fewer PPCs and better oxygenation in patients undergoing abdominal surgery.

## 1. Introduction

During general anaesthesia, progressive atelectasis may develop even in preoperatively healthy lungs [1]. After the induction of anaesthesia and mechanical ventilation, collapsed and overdistended areas of the lungs can develop at the same time [2]. In these scenarios, when lung aeration is inhomogeneous, mechanical ventilation may lead to further tissue damage by inducing biomechanical processes and mechanical stress [2,3].

Protective strategies, such as applying low tidal volumes (VT = 6 mL kg^−1^ of Ideal Body Weight, IBW) and appropriate PEEP levels combined with repeated alveolar recruitment manoeuvres during general anaesthesia are potential tools for lung-protective ventilation [4,5,6,7,8,9] by preventing atelectasis, thus resulting in improved respiratory mechanics and oxygenation [4,10,11].

Due to the disposition of the diaphragm and the loss of tone of the respiratory muscles, the relationship between functional residual capacity and closing capacity changes, and progressive atelectasis develops. During mechanical ventilation, an immediate decrease in pulmonary compliance is often observed that progressively decreases during general anaesthesia [3,9].

However, uniform settings may not suit everyone; hence, several efforts for individualising PEEP have been tried and tested over the years [4,12,13,14,15]. The main target parameters currently used for titration are lowest driving pressure (dP), plateau pressure (Ppl), and transpulmonary pressure (Ptp) or highest pulmonary compliance (static, Cstat; or dynamic, Cdyn). Another way of individualising PEEP and mechanical ventilation is by analysing the visual picture of the lungs by applying electrical impedance tomography or lung ultrasound [14,16]. These imaging techniques provide real-time and mainly continuous information on the ratio of collapsed, distended, or even overdistended areas. After an initial alveolar recruitment manoeuvre (ARM), a decremental PEEP titration method is usually used, and the level of PEEP resulting in the optimal value of the target parameter is considered as the optimal PEEP (PEEPopt).

In this systematic review and meta-analysis, we intended to evaluate the perioperative effects of two different approaches of mechanical ventilation in patients undergoing abdominal surgery: the individualised PEEP titration methods versus conventional settings using fixed PEEP throughout the surgical procedure.

## 2. Materials and Methods

This systematic review was registered in advance on 13 Oct 2021 on PROSPERO (CRD42021282228). We have conducted our research following the Cochrane Handbook’s recommendations for Systematic Reviews of Interventions (Version 6.1) [17] and reported our results according to the Preferred Reporting Items for Systematic Reviews and Meta-Analyses (PRISMA) 2020 Statement [18].

### 2.1. Eligibility Criteria

RCTs comparing the effects of an individually titrated PEEP regardless of titration method (study group, SG) with fixed levels of PEEP or zero PEEP (ZEEP) (control group, CG) conducted on adult patients undergoing abdominal surgery under general anaesthesia were included in our research. Both elective and non-elective, laparoscopic, and open abdominal surgeries (major gastrointestinal, gynaecological, and urological) were accepted, regardless of duration. We excluded trials conducted on paediatric populations (< 18 yrs.) or on patients ventilated for reasons other than abdominal surgery (e.g., acute respiratory distress syndrome, acute hypoxemic respiratory failure, etc.). See the PICO framework in the Supplemental Digital Content, Table S1.

### 2.2. Primary and Secondary Outcomes

Our primary outcome was the incidence of postoperative pulmonary complications (PPCs). The secondary outcome measures were end-of-surgery oxygenation (PaO_2_/FiO_2_ ratio and peripheral oxygen saturation); the PEEPopt (defined as the individual optimal pressure, determined during a titration procedure) values used in the SGs after titration; respiratory mechanical parameters such as dynamic (Cdyn) and static (Cstat) pulmonary compliance; driving and plateau pressure (dP and Ppl); vasopressor requirements in the intraoperative period; duration of surgery and anaesthesia; postoperative inflammatory response (indicated by serum procalcitonin, C-reactive protein, and interleukin levels); length of ICU and hospital stay; and overall mortality. We intended to analyse the following subgroups: laparoscopic vs. open abdominal surgery, recruitment manoeuvre (RM) applied vs. not applied, and obese vs. non-obese patients.

### 2.3. Search Method for Identification of Studies

We performed a systematic search on 14 October 2021 and updated it on 26 April 2024 in four medical databases: MEDLINE (via PubMed), Cochrane Library (CENTRAL), Embase, and Web of Science. We used a predefined search query (see Supplemental Digital Content) in the search engines. There was no filter applied, and there were no language restrictions.

### 2.4. Selection of Included Studies

We used EndNote (EndNote X9, Clarivate Analytics) for the management of the identified records and the selection process. After removing the duplicates both automatically and manually, two independent authors (CS and GS) screened the records for eligibility based on the title/abstract and then on the full text. A Cohen’s kappa coefficient (κ) was calculated at both selection stages. Any disagreements were resolved by a third author (ZM).

### 2.5. Data Extraction

Two authors (CS and GS) independently carried out the data extraction. Any disagreements were resolved by a third author (FD). The data were collected into Excel (Microsoft Corporation, *Microsoft Excel*. Version 16.0. Redmond, WA, USA: Microsoft, 2018) sheets. We extracted the following from the included articles: the first author, year of publication, DOI, country, study design, patient demographics, interventions, and the data and parameters of the predefined outcomes.

### 2.6. Assessment of Methodologic Quality and Risk of Bias

We investigated the risk of bias for all the included studies, following the Cochrane collaboration’s recommendations, ‘revised tool for assessing the risk of bias in randomised trials’ [19]. Three investigators (CS, GS, and CT) independently assessed the quality of the studies. Disagreements were resolved by a fourth author (ZM). The quality assessment of the included studies was performed with Grading of Recommendations, Assessment, Development and Evaluation—Pro, based on the recommendations of the Cochrane Collaboration, and using the GRADEPro Guideline Development Tool.

### 2.7. Measurement of Outcome Data

Our primary outcome, i.e., the incidence of PPCs, is a composite outcome of different lung pathologies that share a common pathophysiology. We collected the reported incidence of PPCs from each study. If the overall incidence was not available, we pooled the number of complications that corresponded with a consensus on PPC definitions [20]. All results were recorded within 7 days. The other primary outcome was the mean or median PEEP value in cm H_2_O set in the CGs and after titration in the SGs.

Secondary outcomes (PaO_2_/FiO_2_ ratio and peripheral oxygen saturation at the end of surgery, and respiratory mechanical parameters [Cdyn, Cstat, dP, and Ppl]) were reported at several timepoints during general anaesthesia. We pooled the data reported at the end of surgery. If data were not reported at this timepoint, we used the intraoperative value nearest to the end of surgery.

### 2.8. Data Synthesis and Analysis

The minimum number of studies for performing the meta-analysis was three. For continuous variables, we used mean ± SD, and we calculated mean differences. If the mean ± SD were not reported in the article, we estimated them from the medians, quartiles, minimums, and maximums using the Luo and Shi methods [21,22]. For dichotomous outcomes, risk ratios (RRs) with 95% confidence intervals (CIs) were determined to describe the difference between the different PEEP strategies. For the pooled results, the exact Mantel–Haenszel method (without continuity correction) was applied to handle zero cell counts [23,24]. We applied the Hartung–Knapp adjustment whenever there were more than five studies available for an outcome [25,26].

If raw data were not available, we contacted the corresponding author. All statistical analyses were performed with R (R Core Team 2021, v4.1.2) using the meta (Schwarzer 2022, v6.2-1) and dmetar (Cuijpers, Furukawa, and Ebert 2020, v0.0.9000) packages [27,28,29].

### 2.9. Assessment of Heterogeneity

Statistical heterogeneity was analysed using the I^2^ statistic and the X^2^ test to acquire probability values; *p* < 0.1 was defined to indicate significance. To estimate the heterogeneity, variance measure τ^2^ was applied, as estimated with the Q profile method. Statistical heterogeneity across trials was assessed by means of the Cochrane Q test and the I^2^ values, where *p* < 0.1 was considered as statistically significant.

### 2.10. Protocol Deviation

We had some minor deviations from our protocol submitted to PROSPERO. First, as an additional secondary outcome, we collected data on the PEEP values used in the SGs. Second, instead of intraoperative values, we collected end-of-surgery values regarding the PaO_2_/FiO_2_ ratio and all respiratory mechanical parameters where this was possible, the reasons being that it was the most common timepoint for recording these data and that it has more clinical relevance as well. Fourth, we did not perform a subgroup analysis on recruitment manoeuvre (RM) applied vs. not applied, due to the heterogeneity of ARM approaches.

## 3. Results

**Figure 1:**
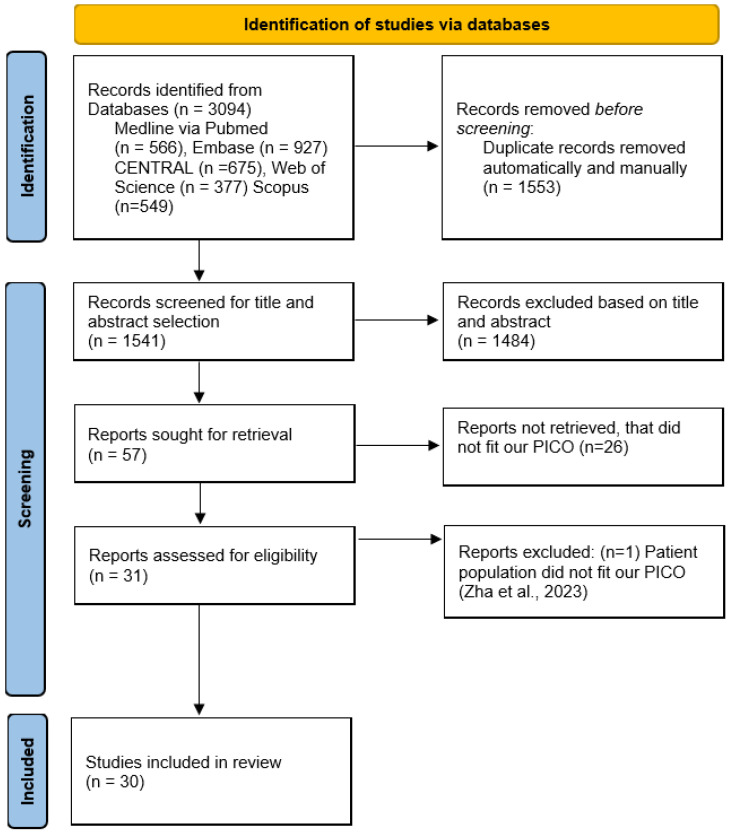
Prisma 2020 Flow Diagram of the screening and selection process [30].

Our systematic search retrieved in 3094 records. After duplicate removal, 1541 articles went through title and abstract selection, during which we had fair agreement (κ = 0.35), whereas after the full-text selection, we had almost perfect agreement (κ = 0.96). We identified 57 RCTs based on title and abstract selection. After full-text selection, we identified 31 studies. After excluding 1 study we found to be ineligible [30], we included 30 studies for quantitative synthesis. The article selection process is depicted in Figure 1.

### 3.1. Characteristics of the Included Studies

The characteristics of the 30 included studies [4,6,9,11,13,14,15,16,31,32,33,34,35,36,37,38,39,40,41,42,43,44,45,46,47,48,49,50,51,52], with a total number of 2602 patients for this systematic review and meta-analysis, are detailed in Table 1.

#### 3.1.1. PEEP Settings

In the SGs, we identified studies that utilised visual based titration methods such as electrical impedance tomography-guided (EIT-guided) [4,14,36,43,52], or lung ultrasound-guided (US-guided) [6,33,40,41]. The other studies determined optimal PEEP by identifying lowest dP [11,37,42,48,49,50], identifying highest Cstat or Cdyn [9,13,15,16,34,38,39,45,46,47,51], or by maintaining a predefined Ptp [15,32,44]. In the CG, PEEP or ZEEP was set by the anaesthetist without any titration.

#### 3.1.2. Types of Surgery

Among the included RCTs, seven [14,32,33,39,46,47,48] were conducted on obese populations undergoing laparoscopic bariatric surgery; other studies were conducted on non-obese patients undergoing either only laparoscopic [6,11,16,31,36,37,38,40,41,43,44,50] or open surgery [9,34,39,42,45,49]. The remaining studies were categorised as miscellaneous because they had mixed populations or lacked sufficient data for identifying a specific category [4,13,15,35,51].

### 3.2. Primary Outcome

#### Postoperative Pulmonary Complications (PPCs)

**Figure 2:**
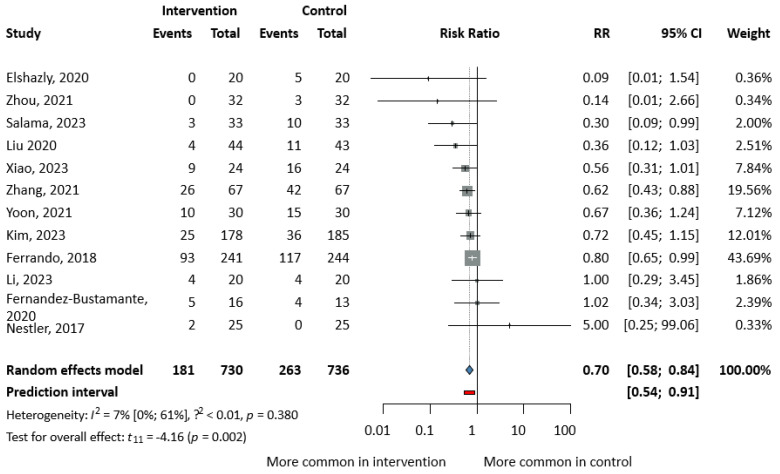
Forest plot of studies representing the incidence of postoperative pulmonary complications. For the version with subgroups, see Figure S12 [13,14,15,16,33,37,39,40,45,49,51,52].

Pooling reported PPCs (atelectasis, pneumonia, ARDS, or pulmonary aspiration, alone or in combination) from 12 studies [13,14,15,16,33,37,39,40,45,49,51,52] with a total of 1466 subjects, 444 had suffered from PPCs. The incidence of PPCs was significantly lower in the SG as compared to the CG (24.8% vs. 35.7%, RR = 0.70, CI: 0.58–0.84, I^2^ = 7%, *p* = 0.002) as shown in Figure 2.

### 3.3. Secondary Outcomes

#### 3.3.1. PaO_2_/FiO_2_ at the End of Surgery

**Figure 3:**
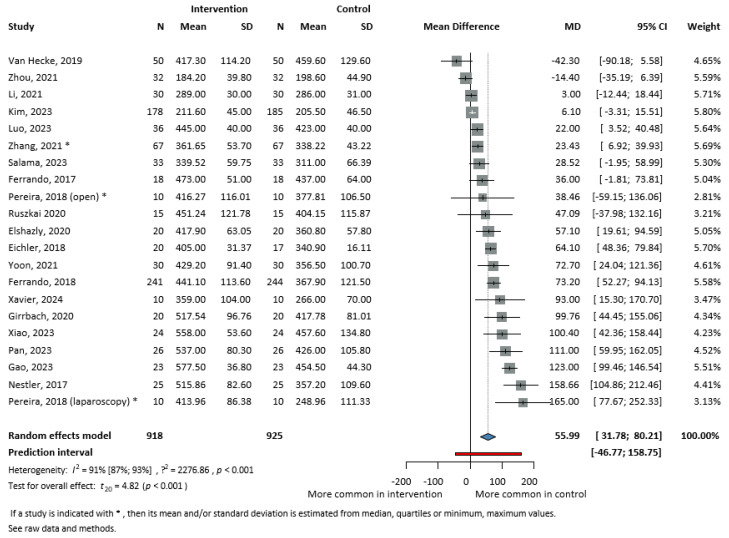
Forest plot of the mean difference in PaO_2_/FiO_2_ ratio at the end of surgery. For the version with subgroups, see Figure S13 [4,9,13,14,16,32,33,34,35,36,37,41,43,45,46,47,49,51,52,53].

Twenty RCTs [4,9,13,14,16,32,33,34,35,36,37,38,41,43,45,46,47,49,51,52] (1843 patients) showed that patients in the SG had significantly higher PaO_2_/FiO_2_ values at the end of the surgery, as compared to those who were in the CG (MD = 55.99 mmHg, 95% CI: 31.78–80.21, I^2^ = 91%, *p* < 0.001) presented in Figure 3.

#### 3.3.2. Titrated PEEP Values in the SGs

**Figure 4:**
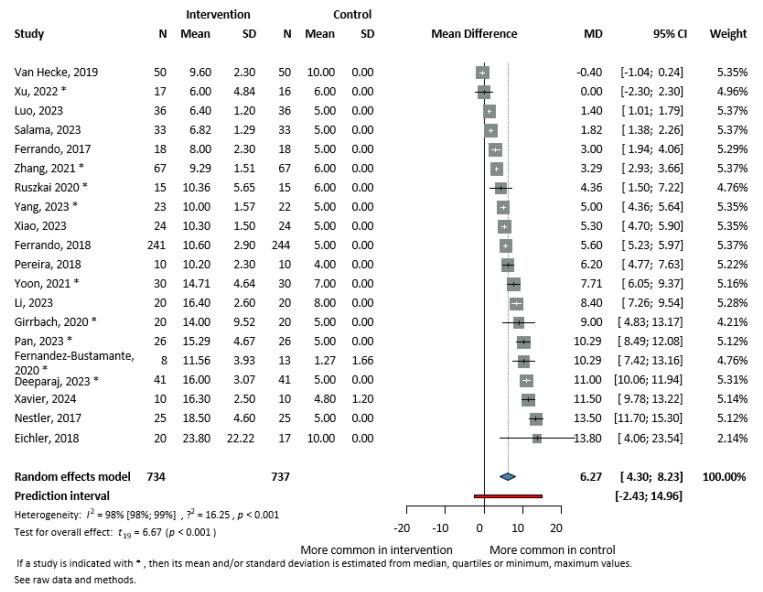
Forest plot presenting mean PEEP values in cm H_2_O. For the version with subgroups, see Figure S13 [4,9,11,13,14,15,16,31,32,34,36,39,41,43,45,46,47,48,49,52].

Twenty studies [4,9,11,13,14,15,16,31,32,34,36,39,41,43,45,46,47,48,49,52] (1471 patients) reported the mean of the utilised PEEP values. In the SG, the level of PEEPopt was more than 6 cm H_2_O higher than the predefined (fixed) value applied in the CG (MD = 6.27 cm H_2_O, CI: 4.30–8.23, I^2^ = 98.0%, *p* ≤ 0.001) (Figure 4). In the SGs, the highest mean PEEPopt was 23.8 cm H_2_O [32] and the lowest was 6.00 (4.84) cm H_2_O [11], creating also the lowest MD if not considering the only case when the fixed PEEP was higher on average [46].

#### 3.3.3. Vasopressor Requirement

Fourteen studies [4,11,13,14,15,16,35,36,38,41,46,49,50,51] (1261 patients) reported data on the number of patients requiring vasopressor support (Supplemental Digital Content, Figure S2). More patients were given vasopressors in the SG than in the CG (58.9% vs. 54.7%). The overall risk ratio of the pooled data showed higher risk of receiving vasopressor support in the SG, although it did not reach the level of significance (RR = 1.07, 95% CI: 1.00–1.14, I^2^ = 0%, *p* = 0.062).

Four studies [9,14,32,36] (157 patients) reported data on maximum norepinephrine doses, showing no significant differences between groups (MD = −0.19 mcg/min/kg 95% CI: −2.40–2.01, I^2^ = 90%, *p* = 0.797) or subgroups (Figure S3). No significant differences were found in total use of either ephedrine (326 patients, MD = 0.22 mg 95% CI: −1.23–1.68, I^2^ = 70%, *p* = 0.710) or phenylephrine (416 patients, MD = 0.00 mcg 95% CI: −0.00–0.00, I^2^ = 0%, *p* = 0.590) [15,16,35,39,41,43,49,51] (Figures S4 and S5).

#### 3.3.4. Respiratory Mechanics

Eleven studies [16,33,34,35,37,38,39,41,43,46,52] (917 patients) reported Cdyn, and another eleven [4,9,14,16,31,39,42,44,45,49,50] showed data on Cstat (656 patients) at the end of surgery. While Cdyn only showed a tendency to improve, Cstat was significantly higher in the SG as compared to the CG (Cdyn: MD = 3.26 mL/cm H_2_O 95% CI: −0.08–6.61, I^2^ = 96%, *p* = 0.055; Cstat: MD = 11.92 mL/cm H_2_O 95% CI: 6.40–17.45, I^2^ = 85%, *p* < 0.001, respectively) as shown in the Supplemental Digital Content in Figure S6A,B). Driving pressure (dP) at the end of surgery was reported in 15 studies [4,9,13,14,16,34,36,37,42,43,44,45,47,49,52] including 1530 patients (Supplemental Digital Content, Figure S7A). The mean dP value was significantly lower in the SG as compared to the CG (MD = −2.75 cm H_2_O, 95% CI: −3.95 to −1.55 I^2^ = 89%, *p* < 0.001). Plateau pressure (Ppl) was measured in 18 studies [4,13,14,16,31,33,36,37,39,41,42,43,44,45,49,50] (1762 patients), and values were significantly higher in the SG as compared to the CG at the end of surgery (MD = 2.49 cm H_2_O 95% CI: 1.08–3.90 I^2^ = 92%, *p* = 0.002) presented in the Supplemental Digital Content (Figure S7B).

#### 3.3.5. Duration of Anaesthesia and Surgery

Regarding the duration of anaesthesia (19 RCTs [4,6,9,11,13,15,16,32,37,38,39,40,41,43,44,48,49,50,52], 1822 patients) and duration of surgery (24 studies [4,6,6,9,11,13,15,16,32,34,35,37,38,40,41,42,43,44,45,47,48,49,50,51,52,52], 2096 patients), there was a slightly longer procedure time in the SG that only reached statistical significance in the latter outcome. As compared to the CG, the duration of anaesthesia and surgery were about 1 (MD = +0.49 min, 95% CI: −6.08–7.06 I^2^ = 62%, *p* = 0.877) and 5 min (MD = + 4.82 min, 95% CI: −2.84–6.81, I^2^ = 23%, *p* < 0.001) longer in the SG (Figure S8A,B).

#### 3.3.6. Length of Hospital Stay, Length of ICU Stay, and Mortality

No significant differences were detected in the length of hospital stay (14 studies [4,6,9,13,15,16,37,40,42,44,45,46,49,51], 1699 patients) or in the length of ICU stay (4 studies, 626 patients) among the study groups (MD = −0.06 days, 95% CI: −0.71–0.59, I^2^ = 71.0%, *p* = 0.855, and MD = −0.10 days, 95% CI: −2.70–2.51, I^2^ = 77%, *p* = 0.914) (Supplemental Digital Content, Figure S9A,B). Mortality was recorded in five studies [6,13,14,45,49] totalling 850 patients (Supplemental Digital Content, Figure S10). Six patients died within 28 days, and the overall RR showed no significant difference between the groups (RR = 1.0, 95% CI: 0.41–2.46 I^2^ = 0%, *p* = 0.9911).

#### 3.3.7. Outcomes with Insufficient Reporting

Except for four studies [13,16,33,45] including 651 patients that reported a slightly higher oxygen saturation at the end of surgery (MD = +0.48%, 95% CI: 0.36–0.61, I^2^ = 0%, *p* = 0.001), this outcome was under-reported to perform a subgroup analysis (Figure S11). We had insufficient data on postoperative IL 6 [15,38], C-reactive protein (CRP) [16], and procalcitonin (PCT) [9] values.

#### 3.3.8. Risk of Bias and Quality Assessment

Three authors (CS, CT, and GS) independently used the Cochrane Collaboration Risk of Bias tool as part of the quality assessment. Any disagreements were resolved by a fourth author (FD). The assessors suggested ’some risk’ for the majority of the studies (19) and a low risk of bias for the rest (11). The risk of bias and a summary-of-findings table of the quality assessment (GRADE) of the included studies can be found in the Supplemental Digital Content (Figure S1, Table S2).

## 4. Discussion

The aim of our systematic review and meta-analysis was to investigate and evaluate the effects of individualised PEEP settings versus fixed PEEP values applied during abdominal surgery on perioperative outcomes, and we found that the PEEP being titrated individually resulted in fewer PPCs, increased oxygenation (PaO_2_/FiO_2_), higher PEEP levels, and better respiratory mechanics. However, we detected a tendency toward a higher vasopressor requirement when individualised PEEP was applied.

### 4.1. Positive End-Expiratory Pressure Setting

Our findings correlate with the results of previous studies [53,54]. In general, appropriate PEEPopt in the SG was 6,27 cm H_2_O higher on average than the mean PEEP values in the CG, indicating higher individual needs than recommended (5–6 cm H_2_O). This difference was even more obvious in the obese subgroup (MD: 8.16 cm H_2_O higher) with a higher risk of developing atelectasis during mechanical ventilation. Obese patients required higher levels of PEEPopt (mean PEEPopt values of this subgroup ranging between 9.6 and 23.8 cm H_2_O) to prevent atelectasis and to improve oxygenation (Figure S12). These settings were higher than the settings in the PROBESE study, where there were no differences found in most of the intra- and postoperative outcomes [55].

We found only one study in this subgroup where PEEPopt was not higher in the SG. However, predefined PEEP in the CG in this trial was relatively high (10 cm H_2_O) and at least higher than usual. Nevertheless, this study was not shown to be an outlier statistically [46].

The mean PEEPopt never exceeded 16 cm H_2_O in the non-obese population. However, regarding subgroups, it was higher during laparoscopic surgery as compared to open procedures (MD: 6.52 cm H_2_O vs. 3.91 cm H_2_O, Figure S12), indicating the adverse effects of pneumoperitoneum that had to be compensated by elevated intraabdominal pressure on respiratory mechanics. A recent physiological study also confirmed that patients undergoing laparoscopic surgery in the Trendelenburg position required higher levels of PEEP in the individualised group compared to those in the fixed (5 cm H_2_O) PEEP group [56]. Previous studies comparing higher and lower PEEP levels did not show clear benefits of using higher PEEP [55,57].

### 4.2. Lung-Function-Related Outcomes

Our main outcome was the incidence of PPCs. According to a consensus recommendation in 2018 [20], PPC was defined as the occurrence of atelectasis, pneumonia, ARDS, or pulmonary aspiration, alone or in combination. Unfortunately, the timeframe within which PPCs should be assessed was not identified. Nevertheless, after abdominal surgeries, PPCs are frequent and are associated with increased morbidity and mortality, as well as prolonged length of hospital stay [58,59,60]. Therefore, the prevalence of PPC has an important potential impact on both patient recovery and health care costs [59,61]. Applying the appropriate level of PEEP as part of lung-protective mechanical ventilation (LPV) may reduce the incidence of PPCs [62,63]. A previous study on patients undergoing abdominal surgery showed significantly fewer PPCs among the group ventilated with LPV applying a median PEEP of 6 cm H_2_O compared to patients ventilated with a non-LPV approach using ZEEP (17.5 vs. 36.0%) [63].

Although the risk of PPCs was higher in our meta-analysis (SG: 24.8%, CG: 35.7%), our results also found a 30% lower risk of developing PPCs in the SG. This is less than was found of a previous meta-analysis of patients undergoing thoracic surgery with a reported RR of 0.52 in the individualised group [53]. The findings of the latest published meta-analysis on patients undergoing abdominal surgery align with our results, showing a lower risk of PPCs in the individualised group with a RR of 0.69 [54].

The mean PaO_2_/FiO_2_ ratio was higher at the end of surgery in the SG in all studies except for two [46,51]. Our results not only show a significantly higher overall MD of PaO_2_/FiO_2_ ratios in the SG as compared to the CG, but this was even higher than detected in the previously mentioned meta-analyses including patients undergoing thoracic (MD = 37.72 mmHg) and abdominal surgery (MD = 20.8 mmHg) [53,54]. A recent meta-analysis comparing EIT-guided PEEP titrations vs. fixed PEEP also found significantly better oxygenation intraoperatively, with an oxygenation index more than 90 mmHg higher among patients with individualised PEEP [64].

Higher PEEP increases the afterload and may decrease the preload of the right ventricle, which can potentially lead to a reduction in cardiac output. Therefore, PEEP should always be applied with caution in haemodynamically unstable patients, particularly in those with hypovolemia [65,66].

We observed a tendency for an increased incidence of vasopressor use in the SG. In a previous study of an obese population, patients receiving individually titrated PEEP required more vasoactive agents during ARMs as compared to fixed PEEP settings of 12 cm H_2_O (92% vs. 48%) [67]. The most profound difference regarding the incidence of vasopressor use was seen in a study of robot-assisted radical prostatectomy, where more patients received vasopressor support in the SG group (90.0% vs. 56.7%; *p* = 0.004) [16].

The overall RR in our study showed a 1.07 times higher risk of not receiving vasopressor support in the CG. However, our result was not significant. Four studies [9,14,32,36] reported data on norepinephrine supply rate, out of which only one [32] showed lower mean dosing in the SG. Due to the lack of sufficient amount of data on vasopressor use, drawing conclusions on the effect of individualised PEEP titration is questionable. A recent physiological trial found no difference in the maximal norephinephrine infusion rate between individualised and a fixed PEEP strategies [56].

### 4.3. Respiratory Mechanics

Our results indicated better lung compliance in both Cdyn and Cstat in the SG, with a significant increase in the latter. Increased pulmonary compliance is common under some pathological conditions; however, except for one trial [41], the studies included in our meta-analysis were conducted on patients without known lung pathology. It is important to note that the most frequently applied strategy in finding PEEPopt was to aim for the highest pulmonary compliance (Cstat or Cdyn); hence, some selection bias could not be excluded, and data on compliance in some of these studies were not reported. Boesing et al. found that when comparing two individualised methods, both were effective in reducing dynamic lung strain and driving pressure compared to a fixed PEEP of 5 cm H_2_O [56]. Regarding the question of whether it is the higher or lower PEEP or the personalisation that truly matters, another study comparing individualised methods found significantly better respiratory mechanics and oxygenation with Peso-guided titration than with gas-exchange-led titration [68].

In a recently published RCT, PEEP was regularly re-evaluated after the primary titration method during the entire surgical procedure. This has some rationale, as a progressive decrease in lung compliance during mechanical ventilation lasting for several hours is a well-known phenomenon [11].

Excessive driving pressure and elevated plateau pressure are known risk factors of ventilator-induced lung injury [69]. Therefore, it is understandable why optimising dP was the method of choice for titrating PEEPopt in six studies [11,37,42,48,49,50]. In a previous study, secondary analysis was performed on obese patients that compared three PEEP settings: individualised, fixed PEEP of 12 cm H_2_O, and fixed PEEP of 4–5 cm H_2_O. They found the lowest mean intraoperative dP in the individualised PEEP group (9.8 cm H_2_O vs. 14.4 cm H_2_O vs. 18.8 cm H_2_O, *p* < 0.001) [67]. This may suggest that using an individualised approach is better than applying a higher PEEP alone.

Previous studies found repeated recruitment manoeuvres (ARM) to be beneficial during general anaesthesia as part of lung-protective ventilation [9,63,70,71,72]. However, we did not observe better outcomes among the studies that used ARMs regularly or at least occasionally compared to those that did not.

### 4.4. Other Outcomes

The duration of anaesthesia was not prolonged significantly in the SG. The duration of surgery showed a statistically significant prolongation with less then 5 min; however, its clinical relevance is questionable. These findings suggest that the intervention is not significantly time-consuming. We did not find any significant differences regarding the length of hospital or ICU stay, suggesting that individualising PEEP does not seem to affect the length of these outcomes. Only a few studies reported data on mortality. This is not surprising, as mortality in this patient population is very low. In a meta-analysis of 3.6 million patients undergoing bariatric surgery, only 4707 perioperative deaths were reported [73]. In another study of more than 35 000 patients undergoing radical prostatectomy, the reported 30-day mortality was less than 0.5% [74]. Therefore, the feasibility of a well-powered study on mortality in these patient populations is questionable.

### 4.5. Strengths and Limitations

To the best of our knowledge, our meta-analysis on the effects of individualised PEEP titration compared to conventional PEEP settings in patients undergoing abdominal surgery is the most recent one and includes the highest number of patients to date. Furthermore, we only included randomised controlled trials, and in order to be transparent, we carried out our systematic review and meta-analysis following our protocol submitted to PROSPERO in advance, from which we made only minor deviations.

Our review has several limitations. First, there was a heterogeneity of the included population regarding the age, gender, and type of surgery; the titration methods used in the SG also differed as well as in CGs different PEEP settings were applied. Second, the measurements of some of our outcome data differed in divisions or timepoints. Third, there was heterogeneity regarding the definitions of PPC, which we intended to solve by following a recommendation of definitions of PPCs [20]. Fourth, we had very limited data on certain outcomes such as vasopressor need; hence, we could not make any feasible comment on the relationship between PEEP titration and its haemodynamic consequences. Furthermore, our study cannot answer whether it is the individualised approach per se that is beneficial or that the commonly used PEEP of around 5–6 cm H_2_O is inappropriate. Therefore, individualised PEEP titration should be tested against a higher preset PEEP (i.e., 10 cm H_2_O). Finally, we were unable to present data on postoperative pulmonary complications that could potentially have arisen from the higher PEEP used, as this aspect was under-reported across studies.

## 5. Conclusions

Based on our findings, individualised PEEP titration significantly reduces the risk of PPCs and results in better oxygenation as compared to a conventionally applied fixed PEEP strategy. Individualised PEEP may also lead to better lung mechanics. Regarding the implications of our results, further research should be conducted on a more homogenous population, with a consensus on titration methods, definitions, and timepoints of outcome measurements and different preset PEEP levels.

## Figures and Tables

**Table 1:** Characteristics of the included studies.

Study	Patients–Intervention/Control (*n*)	Type of Surgery	Study Group Titration Method	Control Group PEEP Used (cm H_2_O)
Deeparaj et al., 2023 [31]	41/41	laparoscopic gynaecological surgery	Cstat ^1^-guided	5
Eichler et al., 2018 [32]	20/17	laparoscopic bariatric surgery	Ptp ^2^-guided	10
Elshazly et al., 2021 [33]	20/20	laparoscopic bariatric surgery	US ^3^-guided	4
Fernandez-Bustamante et al., 2020 [15]	24/13	laparoscopic and open abdominal surgeries	Cstat- or Ptp-guided	≤2
Ferrado et al., 2017 [34]	18/18	open abdominal surgery	Cdyn ^4^-guided	5
Ferrado et al., 2018 [13]	241/244	laparoscopic and open abdominal surgeries	Cdyn-guided	5
Gao et al., 2023 [35]	23/23	robotic-assisted laparoscopic prostatectomy	FiO_2_-guided ^5^	5
Girrbach et al., 2020 [36]	20/20	robot-assisted laparoscopic radical prostatectomy	EIT ^6^-guided	5
Kim et al., 2023 [37]	178/185	laparoscopic/robotic abdominal surgery	dP ^7^-guided	5
Li et al., 2021 [38]	60/60	laparoscopic surgery	Cdyn-guided	5
Li et al., 2023 [39]	20/20	laparoscopic bariatric surgery	Cdyn-guided	8
Liu et al., 2019 [6]	58/57	laparoscopic radical gastrectomy	US-guided	ZEEP
Liu et al., 2020 [40]	44/43	laparoscopic total hysterectomy	US-guided	ZEEP
Luo et al., 2023 [41]	36/36	laparoscopic gastrointestinal surgery	US-guided	5
Mini et al., 2021 [42]	41/41	open abdominal surgery	dP-guided	5
Nestler et al., 2017 [14]	25/25	laparoscopic bariatric surgery	EIT-guided	5
Pan et al., 2023 [43]	26/26	robot-assisted prostate surgery	EIT-guided	5
Pereira et al., 2018 [4]	20/20	laparoscopic and open abdominal surgeries	EIT-guided	4
Piriyapatsom et al., 2020 [44]	22/22	laparoscopic gynaecological surgery	Ptp-guided	5
Ruszkai et al., 2021 [9]	15/15	open radical cystectomy	Cstat-guided	6
Salama et al., 2023 [45]	33/33	open abdominal surgery	Cstat-guided	5
Van Hecke et al., 2019 [46]	50/50	laparoscopic bariatric surgery	Cdyn-guided	10
Xavier et al., 2024 [47]	10/10	laparoscopic bariatric surgery	Cstat-guided	5
Xiao et al., 2023 [52]	24/24	CRS + HIPEC	EIT-guided	5
Xu et al., 2022 [11]	17/16	laparoscopic surgery	dP-guided	6
Yang et al., 2023 [48]	23/22	laparoscopic sleeve gastrectomy	dP-guided	5
Yoon et al., 2021 [16]	30/30	robot-assisted radical prostatectomy	Cdyn-guided	7
Zhang et al., 2021 [49]	67/67	open upper abdominal surgery	dP-guided	6
Zhang et al., 2022 [50]	67/67	laparoscopic gynaecological surgery	dP-guided	5
Zhou et al., 2021 [51]	32/32	laparoscopic robot-assisted prostatectomy	Cdyn-guided	ZEEP

^1^ Cstat: static compliance. ^2^ Ptp: transpulmonary pressure. ^3^ US: ultrasound. ^4^ Cdyn: dynamic compliance. ^5^ FiO_2_ fraction of inspired oxygen. ^6^ EIT: electronic impedance tomograph. ^7^ dP: driving pressure.

## Data Availability

The dataset supporting the conclusions of this article is available from the corresponding author.

## Supplementary Materials

The following supporting information can be downloaded at https://www.mdpi.com/article/10.3390/jcm13226776/s1, Figure S1: Results of the risk of bias assessment, using RoB-2 Tool. Figure S2: Number of patients who needed vasopressor support. Figure S3: Maximal dose of norepinephrine. Figure S4: Total amount of ephedrine used (mg). Figure S5: Total amount of fenilephrine used (ug). Figure S6A: Dynamic compliance (Cdyn) in mL/cm H_2_O, at the end of the surgery. Figure S6B: Static compliance (Cstat) in mL/cm H_2_O, at the end of the surgery. Figure S7A: Driving pressure in cm H_2_O, at the end of the surgery. Figure S7B. Plateau pressure in cm H_2_O, at the end of the surgery. Figure S8A: Duration of anaesthesia in minutes. Figure S8B: Duration of surgery in minutes. Figure S9A: Length of hospital stay in days. Figure S9B: Length of ICU stay in days. Figure S10: Mortality. Figure S11: SpO2 measured at the end of the surgery. Figure S12: PEEP values, subgroup analysis. Figure S13: PPCs, subgroup analysis. Figure S14: PaO_2_/FiO_2_ ratio, subgroup analysis. Figure S15: Search strategy used in the four medical databases on 14 October 2021 and 26 April 2024. Table S1: PICO framework. Table S2: GRADE Assessment: summary of findings table.
