# Supplementary material for: Individualised Positive End-Expiratory Pressure Settings Reduce the Incidence of Postoperative Pulmonary Complications: A Systematic Review and Meta-Analysis
Source: J Clin Med. 2024 Nov 11;13(22):6776. doi: 10.3390/jcm13226776 (PMC11595123; doi:10.3390/jcm13226776)
Supplement: Supplementary file 1 [file jcm-13-06776-s001.zip › jcm-3274902-supplementary.pdf]

Individualised Positive End-Expiratory Pressure Settings Reduce the Incidence of Postoperative Pulmonary Complications - **Supplemental Digital Content**

Supplemental Digital Content, Figure S1 Results of the risk of bias assesment, using RoB-2 Tool

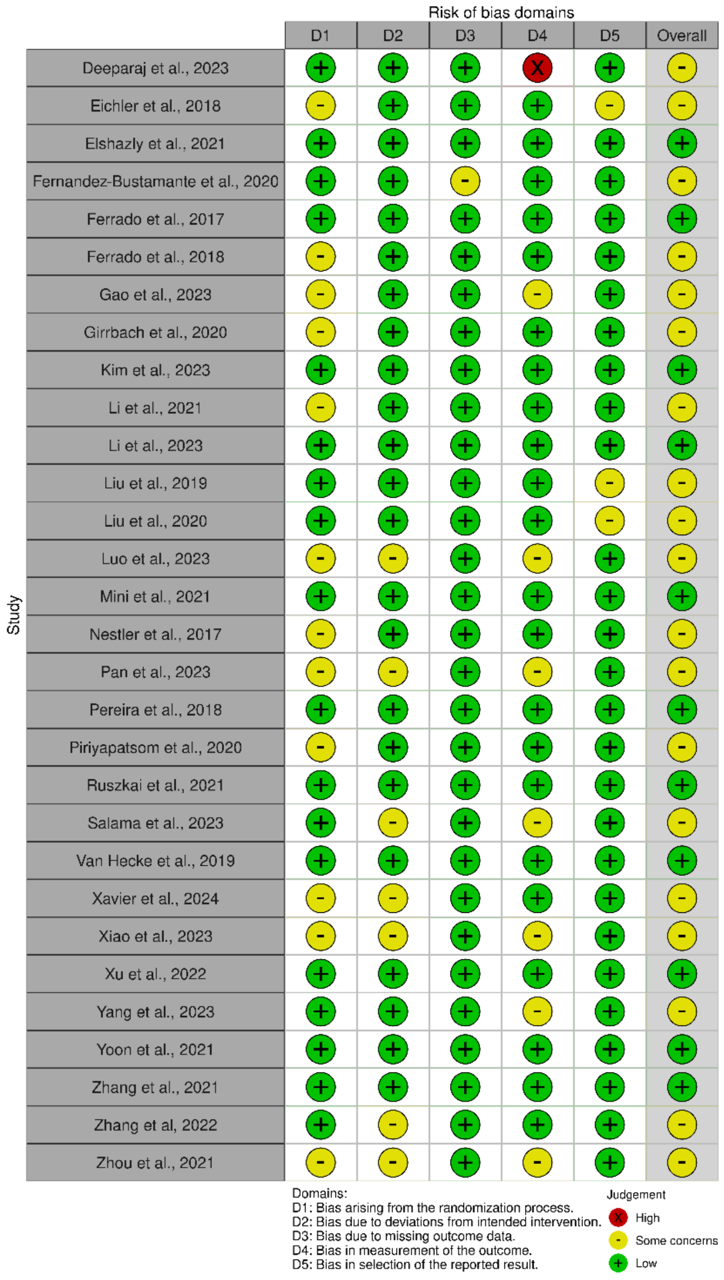

.

Supplemental Digital Content, Figure S2. Number of patients who needed vasopressor support.

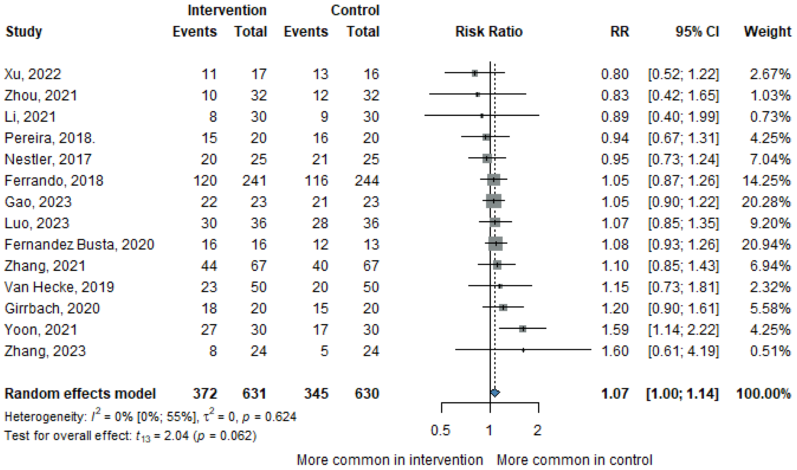

Supplemental Digital Content, Figure S3 – Maximal dose of norepinephrine in mcg/kg/min

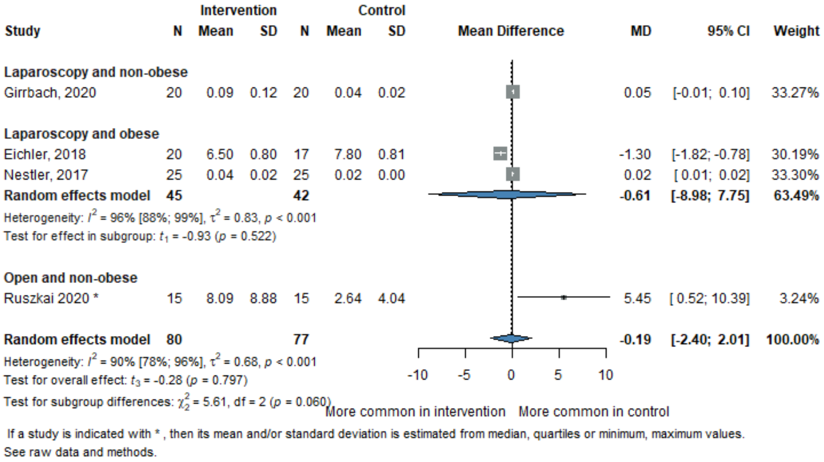

Supplemental Digital Content,Figure S4 – Total amount of ephedrine used (mg)

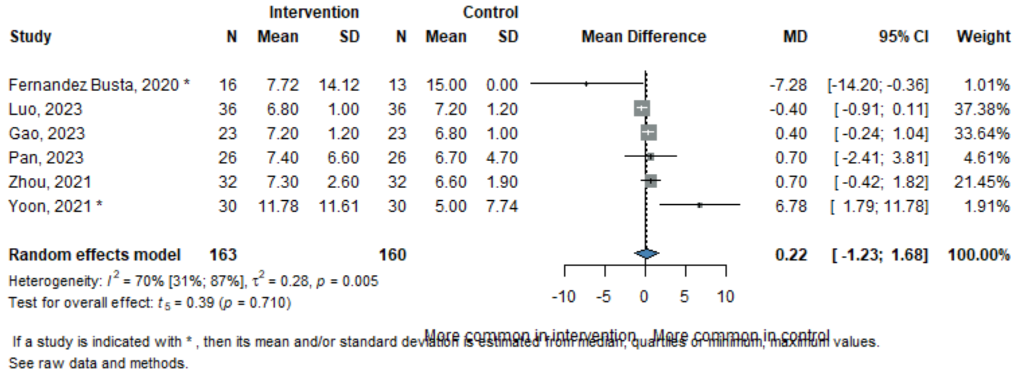

Supplemental Digital Content,Figure S5 – Total amount of fenilephrine used (ug)

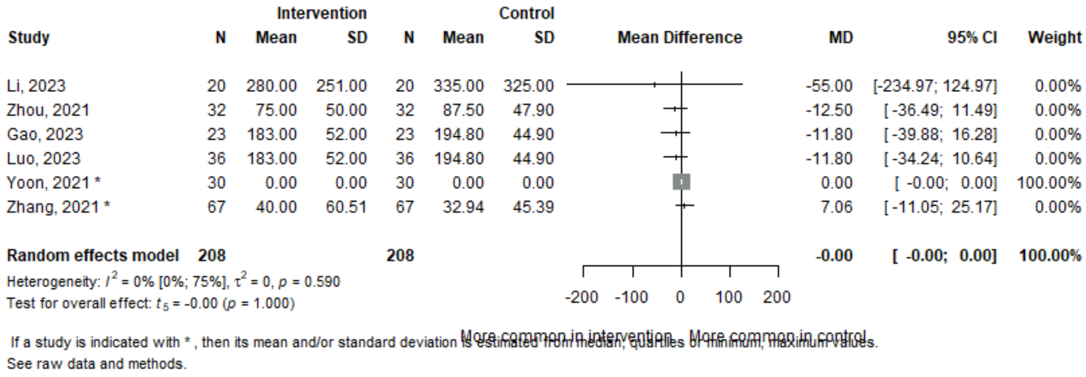

Supplemental Digital Content,Figure S6A Dynamic compliance (Cdyn) in ml/cm H_2O, at the end of the surgery.

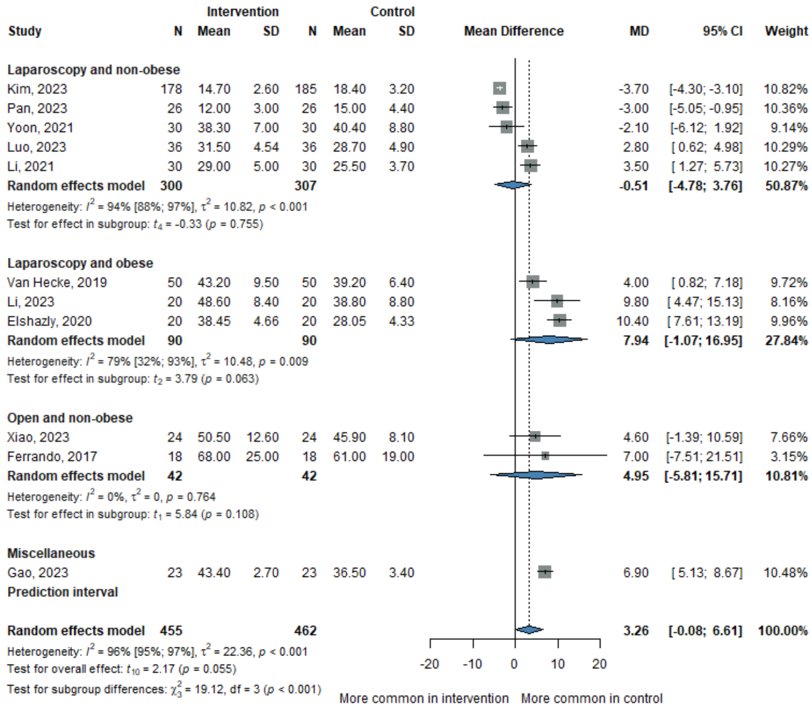

Supplemental Digital Content,Figure S6B Static compliance (Cstat) in ml/cm H_2O, at the end of the surgery.

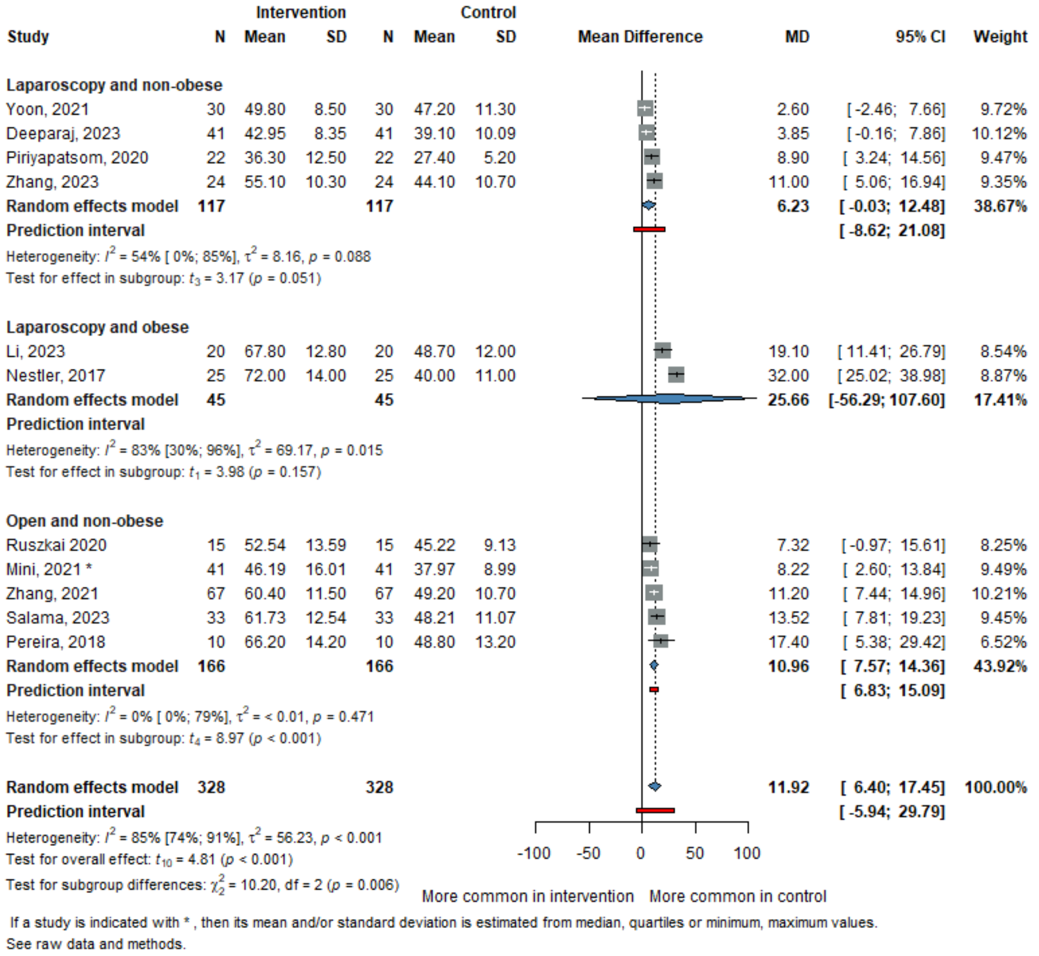

Supplemental Digital Content,Figure S7A Driving pressure in cm H_2O, at the end of the surgery.

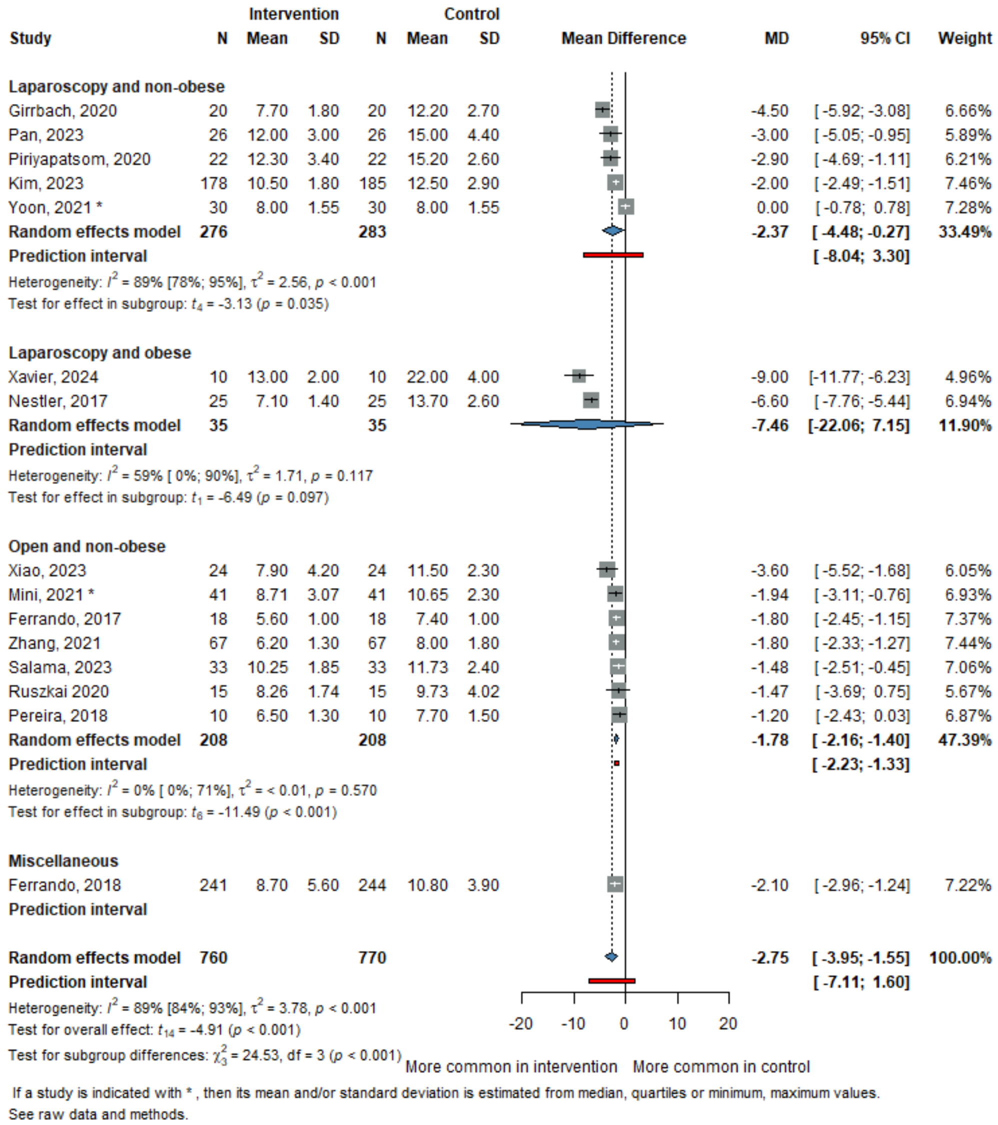

Supplemental Digital Content, Figure S7B. Plateau pressure in cm H_2O, at the end of the surgery.

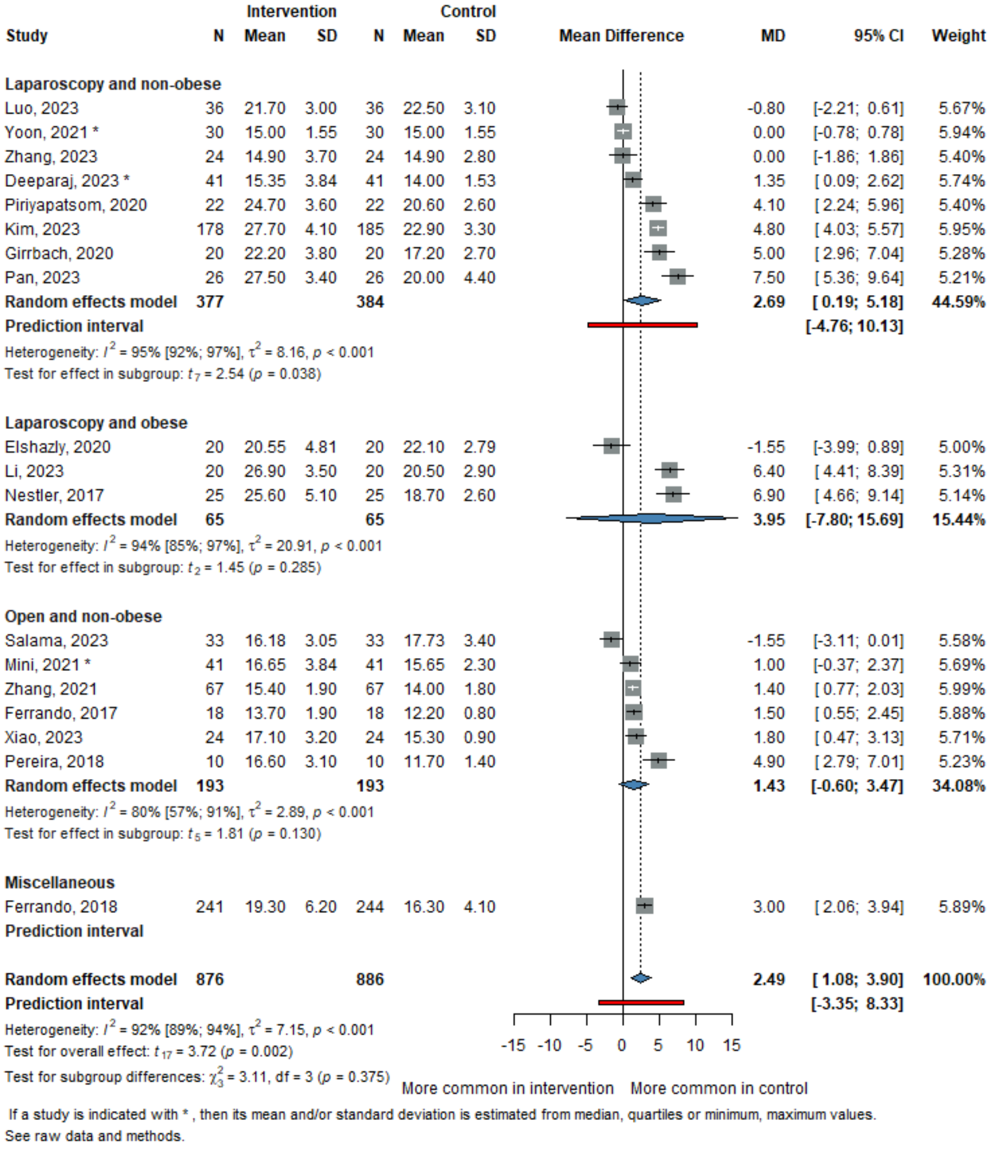

Supplemental Digital Content,Figure S8A Duration of anesthesia in minutes.

Study	Intervention N	Intervention Mean	Intervention SD	Control N	Control Mean	Control SD	MD	95% CI	Weight
**Laparoscopy and non-obese**									
Xu, 2022	17	249.00	65.00	16	269.00	64.00	-20.00	[-64.02; 24.02]	1.60%
Zhang, 2023	24	180.00	68.00	24	197.00	56.00	-17.00	[-52.24; 18.24]	2.35%
Pan, 2023	26	218.00	34.10	26	227.40	35.10	-9.40	[-28.21; 9.41]	5.80%
Luo, 2023	36	218.70	5.60	36	227.30	7.80	-8.60	[-11.74; -5.46]	13.48%
Kim, 2023 *	178	175.78	64.97	185	183.51	59.75	-7.73	[-20.59; 5.12]	8.44%
Piriyapatsom, 2020	22	190.70	81.10	22	191.70	59.30	-1.00	[-42.98; 40.98]	1.74%
Liu 2019	58	323.25	70.83	57	320.77	65.22	2.48	[-22.40; 27.36]	4.03%
Liu, 2020	44	156.15	50.43	43	145.97	60.02	10.18	[-13.14; 33.50]	4.41%
Li, 2021 *	30	130.00	38.70	30	111.29	37.15	18.71	[-0.48; 37.91]	5.67%
Yoon, 2021 *	30	165.26	61.92	30	137.50	19.35	27.76	[4.55; 50.98]	4.44%
**Random effects model**	**465**			**469**			**0.02**	**[-10.28; 10.33]**	**51.97%**
**Prediction interval**								**[-25.56; 25.61]**	
Heterogeneity: $I^2 = 55\%$ [8%; 78%], $\tau^2 = 100.73$, $p = 0.018$									
Test for effect in subgroup: $t_9 = 0.01$ ($p = 0.996$)									
**Laparoscopy and obese**									
Li, 2023	20	142.00	32.00	20	152.00	34.00	-10.00	[-30.46; 10.46]	5.24%
Eichler, 2018	20	200.50	11.04	17	201.90	9.39	-1.40	[-7.98; 5.18]	11.94%
Yang, 2023 *	23	158.23	8.63	22	153.21	11.77	5.02	[-1.03; 11.08]	12.22%
**Random effects model**	**63**			**59**			**0.96**	**[-12.72; 14.63]**	**29.40%**
Heterogeneity: $I^2 = 39\%$ [0%; 81%], $\tau^2 = 11.18$, $p = 0.196$									
Test for effect in subgroup: $t_2 = 0.30$ ($p = 0.792$)									
**Open and non-obese**									
Xiao, 2023	24	710.00	221.70	24	723.80	124.60	-13.80	[-115.55; 87.95]	0.33%
Zhang, 2021	67	258.90	54.60	67	248.40	53.00	10.50	[-7.72; 28.72]	6.02%
Ruszkai 2020	15	418.20	70.49	15	384.00	107.01	34.20	[-30.65; 99.05]	0.79%
**Random effects model**	**106**			**106**			**11.48**	**[-11.28; 34.25]**	**7.14%**
Heterogeneity: $I^2 = 0\%$ [0%; 90%], $\tau^2 = 0$, $p = 0.698$									
Test for effect in subgroup: $t_2 = 2.17$ ($p = 0.162$)									
**Miscellaneous**									
Pereira, 2018. *	20	206.80	51.59	20	234.28	22.22	-27.48	[-52.10; -2.86]	4.09%
Ferrando, 2018 *	241	247.02	89.50	244	235.52	89.49	11.49	[-4.44; 27.42]	6.96%
Fernandez Busta, 2020 *	16	298.73	145.28	13	233.14	93.70	65.59	[-21.94; 153.12]	0.44%
**Random effects model**	**277**			**277**			**3.45**	**[-91.68; 98.57]**	**11.49%**
Heterogeneity: $I^2 = 78\%$ [27%; 93%], $\tau^2 = 854.75$, $p = 0.012$									
Test for effect in subgroup: $t_2 = 0.16$ ($p = 0.890$)									
**Random effects model**	**911**			**911**			**0.49**	**[-6.08; 7.06]**	**100.00%**
Heterogeneity: $I^2 = 62\%$ [37%; 77%], $\tau^2 = 65.13$, $p < 0.001$									
Test for overall effect: $t_{18} = 0.16$ ($p = 0.877$)									
Test for subgroup differences: $\chi^2_3 = 3.40$, df = 3 ($p = 0.334$)									

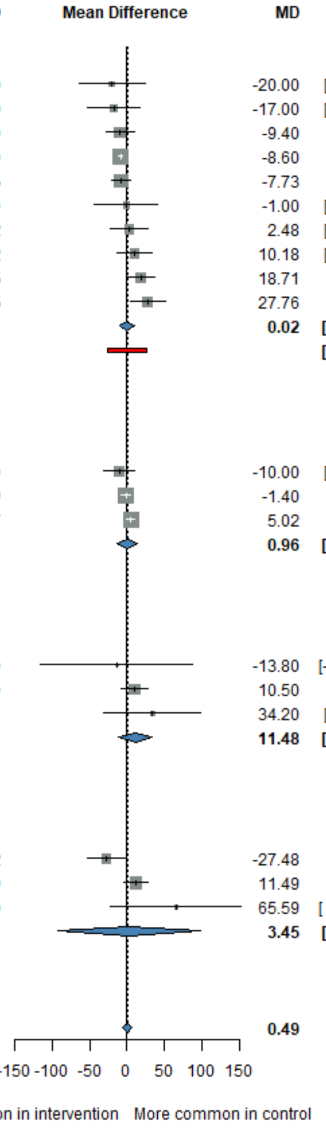

If a study is indicated with * , then its mean and/or standard deviation is estimated from median, quartiles or minimum, maximum values. See raw data and methods.

Supplemental Digital Content, Figure S8B Duration of surgery in minutes.

	Intervention			Control					
Study	N	Mean	SD	N	Mean	SD	MD	95% CI	Weight
**Laparoscopy and non-obese**									
Xu, 2022	17	180.00	113.00	16	203.00	108.00	-23.00	[-98.40; 52.40]	0.05%
Zhou, 2021	32	175.40	52.60	32	183.20	64.70	-7.80	[-36.69; 21.09]	0.33%
Kim, 2023 *	178	141.67	59.00	185	144.40	52.28	-2.73	[-14.22; 8.75]	2.07%
Liu 2019	58	298.95	65.76	57	301.34	54.27	-2.39	[-24.41; 19.63]	0.56%
Pan, 2023	26	169.60	27.40	26	169.40	29.30	0.20	[-15.22; 15.62]	1.15%
Liu, 2020	44	127.00	44.26	43	125.03	34.23	1.97	[-14.63; 18.57]	0.99%
Piriyapatsom, 2020	22	159.80	75.30	22	157.70	56.40	2.10	[-37.21; 41.41]	0.18%
Luo, 2023	36	174.10	4.20	36	169.10	6.40	5.00	[2.50; 7.50]	43.67%
Li, 2021 *	30	92.15	23.22	30	85.21	25.54	6.94	[-5.41; 19.29]	1.79%
Zhang, 2023	24	147.00	65.00	24	139.00	55.00	8.00	[-26.07; 42.07]	0.24%
Yoon, 2021 *	30	127.13	54.18	30	105.35	27.09	21.78	[0.11; 43.46]	0.58%
**Random effects model**	**497**			**501**			**4.60**	**[2.52; 6.67]**	**51.60%**
Heterogeneity: $I^2 = 0\%$ [0%; 60%], $\tau^2 = 0$, $p = 0.790$									
Test for effect in subgroup: $t_{10} = 4.94$ ($p < 0.001$)									
**Laparoscopy and obese**									
Xavier, 2024 *	10	63.87	24.88	10	67.62	17.83	-3.75	[-22.72; 15.22]	0.76%
Yang, 2023 *	23	131.79	11.77	22	130.00	9.42	1.79	[-4.42; 8.01]	7.07%
Eichler, 2018	20	143.30	9.06	17	134.70	8.08	8.60	[3.08; 14.12]	8.95%
**Random effects model**	**53**			**49**			**4.51**	**[-8.21; 17.24]**	**16.77%**
Heterogeneity: $I^2 = 42\%$ [0%; 83%], $\tau^2 = 12.92$, $p = 0.177$									
Test for effect in subgroup: $t_2 = 1.53$ ($p = 0.267$)									
**Open and non-obese**									
Xiao, 2023	24	620.00	210.00	24	622.30	133.30	-2.30	[-101.81; 97.21]	0.03%
Salama, 2023	33	136.36	49.97	33	136.97	64.12	-0.61	[-28.35; 27.13]	0.35%
Zhang, 2021	67	225.80	47.70	67	222.10	54.70	3.70	[-13.68; 21.08]	0.90%
Ferrando, 2017	18	190.00	75.00	18	178.00	86.00	12.00	[-40.71; 64.71]	0.10%
Ruszkai 2020	15	378.00	63.52	15	352.47	103.58	25.53	[-35.96; 87.02]	0.07%
Mini, 2021 *	41	350.32	149.65	41	323.61	170.38	26.71	[-42.71; 96.12]	0.06%
**Random effects model**	**198**			**198**			**5.02**	**[-3.13; 13.17]**	**1.51%**
Heterogeneity: $I^2 = 0\%$ [0%; 75%], $\tau^2 = 0$, $p = 0.957$									
Test for effect in subgroup: $t_5 = 1.58$ ($p = 0.174$)									
**Miscellaneous**									
Pereira, 2018. *	20	140.52	42.06	20	177.48	29.37	-36.96	[-59.45; -14.48]	0.54%
Gao, 2023	23	174.10	4.20	23	169.10	6.40	5.00	[1.87; 8.13]	27.90%
Ferrando, 2018 *	241	209.25	74.58	244	191.75	70.85	17.50	[4.55; 30.45]	1.63%
Fernandez Busta, 2020 *	16	246.41	129.94	13	188.65	76.29	57.76	[-18.23; 133.74]	0.05%
**Random effects model**	**300**			**300**			**2.95**	**[-49.57; 55.47]**	**30.12%**
Heterogeneity: $I^2 = 84\%$ [60%; 94%], $\tau^2 = 742.29$, $p < 0.001$									
Test for effect in subgroup: $t_3 = 0.18$ ($p = 0.870$)									
**Random effects model**	**1048**			**1048**			**4.82**	**[2.84; 6.81]**	**100.00%**
**Prediction interval**								**[3.07; 6.57]**	
Heterogeneity: $I^2 = 23\%$ [0%; 53%], $\tau^2 = 0$, $p = 0.158$									
Test for overall effect: $t_{23} = 5.03$ ($p < 0.001$)									
Test for subgroup differences: $\chi^2_3 = 0.03$, df = 3 ($p = 0.999$)									

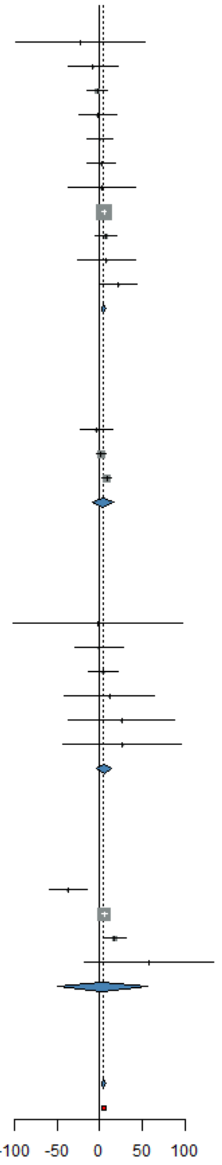

More common in intervention More common in control

If a study is indicated with * , then its mean and/or standard deviation is estimated from median, quartiles or minimum, maximum values. See raw data and methods.

Supplemental Digital Content,Figure S9A Length of hospital stay in days.

Study	Intervention N	Intervention Mean	Intervention SD	Control N	Control Mean	Control SD	MD	95% CI	Weight
**Laparoscopy and non-obese**									
Kim, 2023	178	6.30	4.10	185	7.10	7.10	-0.80	[-1.99; 0.39]	7.44%
Piriyapatsom, 2020	22	2.90	0.70	22	2.90	0.70	0.00	[-0.41; 0.41]	11.30%
Yoon, 2021 *	30	5.00	0.00	30	5.00	0.00	0.00	[-0.00; 0.00]	12.18%
Liu, 2020	44	7.02	2.16	43	6.56	1.23	0.46	[-0.28; 1.20]	9.78%
Zhou, 2021	32	9.30	3.70	32	8.30	3.90	1.00	[-0.86; 2.86]	4.74%
Liu 2019	58	12.60	4.10	57	11.20	3.30	1.40	[0.04; 2.76]	6.63%
**Random effects model**	**364**			**369**			**0.00**	**[-0.00; 0.00]**	**52.06%**
Heterogeneity: I^2 = 41% [0%; 76%], τ^2 = < 0.01, p = 0.134									
Test for effect in subgroup: t_5 = 0.01 (p = 0.996)									
**Laparoscopy and obese**									
Van Hecke, 2019 *	50	3.65	0.76	50	4.00	0.00	-0.35	[-0.56; -0.14]	11.93%
**Open and non-obese**									
Zhang, 2021 *	67	13.00	10.00	67	16.12	10.59	-3.12	[-6.61; 0.37]	1.87%
Ruszkai 2020	15	18.23	11.45	15	20.20	13.08	-1.97	[-10.77; 6.83]	0.34%
Salama, 2023	33	5.50	3.10	33	6.80	4.20	-1.30	[-3.08; 0.48]	5.00%
Mini, 2021 *	41	12.50	3.84	41	13.42	6.14	-0.92	[-3.14; 1.30]	3.78%
**Random effects model**	**156**			**156**			**-1.43**	**[-2.71; -0.15]**	**10.99%**
Heterogeneity: I^2 = 0% [0%; 85%], τ^2 = 0, p = 0.768									
Test for effect in subgroup: t_3 = -3.57 (p = 0.038)									
**Miscellaneous**									
Ferrando, 2018 *	241	7.70	4.47	244	9.05	5.22	-1.35	[-2.22; -0.49]	9.10%
Pereira, 2018. *	20	2.64	0.79	20	2.64	0.79	0.00	[-0.49; 0.49]	10.97%
Fernandez Busta, 2020 *	16	6.36	2.42	13	3.36	2.49	3.00	[1.20; 4.80]	4.94%
**Random effects model**	**277**			**277**			**0.43**	**[-4.95; 5.82]**	**25.01%**
Heterogeneity: I^2 = 90% [72%; 96%], τ^2 = 4.14, p < 0.001									
Test for effect in subgroup: t_2 = 0.35 (p = 0.762)									
**Random effects model**	**847**			**852**			**-0.06**	**[-0.71; 0.59]**	**100.00%**
Heterogeneity: I^2 = 71% [51%; 83%], τ^2 = 0.58, p < 0.001									
Test for overall effect: t_{13} = -0.19 (p = 0.855)									
Test for subgroup differences: χ^2_3 = 23.62, df = 3 (p < 0.001)									

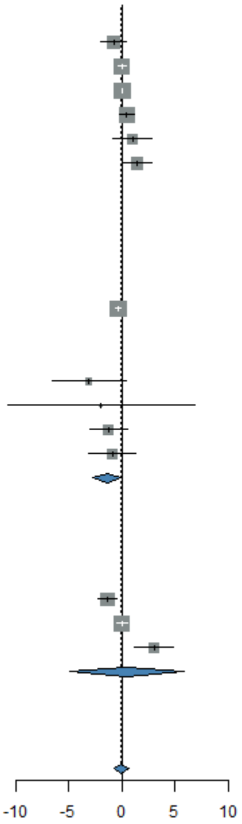

If a study is indicated with * , then its mean and/or standard deviation is estimated from median, quartiles or minimum, maximum values. See raw data and methods.

Supplemental Digital Content, Figure S9B Length of ICU stay in days.

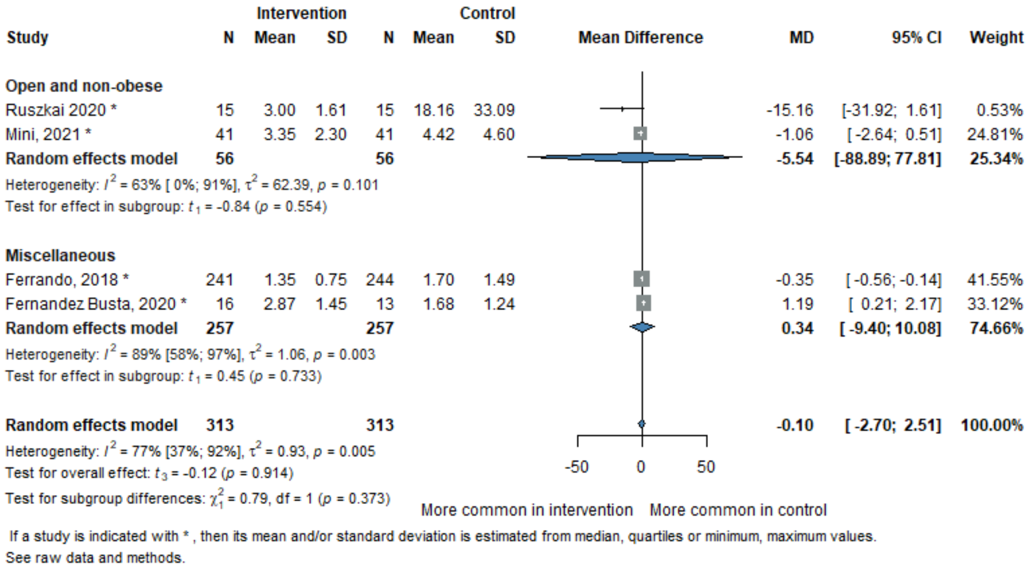

Supplemental Digital Content, Figure S10. Mortality

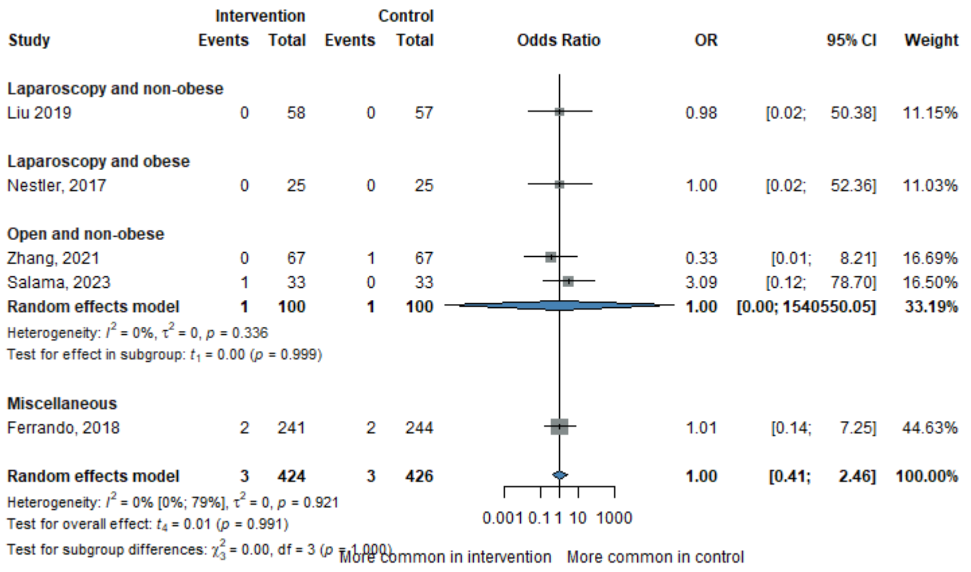

Supplemental Digital Content, Figure S11. SpO2 measured at the end of the surgery

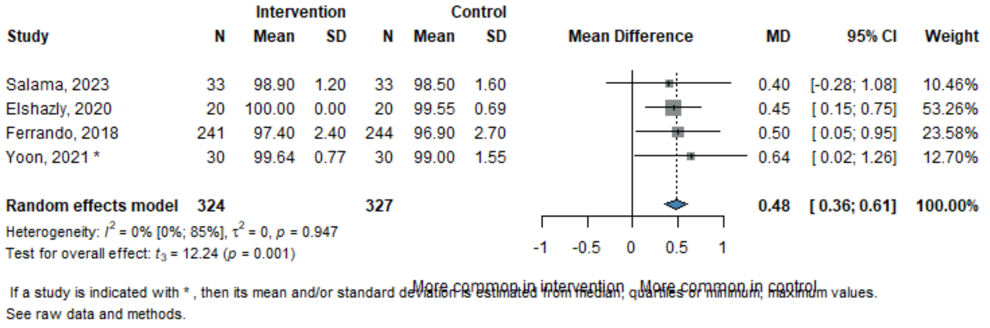

Supplemental Digital Content, Figure S12 – PPCs, subgroup analysis

Study	Intervention Events	Intervention Total	Control Events	Control Total	RR	95% CI	Weight
**Laparoscopy and non-obese**							
Zhou, 2021	0	32	3	32	0.14	[0.01; 2.66]	0.34%
Liu 2020	4	44	11	43	0.36	[0.12; 1.03]	2.51%
Yoon, 2021	10	30	15	30	0.67	[0.36; 1.24]	7.12%
Kim, 2023	25	178	36	185	0.72	[0.45; 1.15]	12.01%
**Random effects model**	**39**	**284**	**65**	**290**	**0.64**	**[0.38; 1.06]**	**21.98%**
Heterogeneity: $I^2 = 0\%$ [0%; 85%], $\tau^2 = 0$, $p = 0.483$							
Test for effect in subgroup: $t_3 = -2.80$ ($p = 0.068$)							
**Laparoscopy and obese**							
Elshazly, 2020	0	20	5	20	0.09	[0.01; 1.54]	0.36%
Li, 2023	4	20	4	20	1.00	[0.29; 3.45]	1.86%
Nestler, 2017	2	25	0	25	5.00	[0.25; 99.06]	0.33%
**Random effects model**	**6**	**65**	**9**	**65**	**0.79**	**[0.01; 69.84]**	**2.55%**
Heterogeneity: $I^2 = 48\%$ [0%; 85%], $\tau^2 = 1.91$, $p = 0.149$							
Test for effect in subgroup: $t_2 = -0.23$ ($p = 0.840$)							
**Open and non-obese**							
Salama, 2023	3	33	10	33	0.30	[0.09; 0.99]	2.00%
Xiao, 2023	9	24	16	24	0.56	[0.31; 1.01]	7.84%
Zhang, 2021	26	67	42	67	0.62	[0.43; 0.88]	19.56%
**Random effects model**	**38**	**124**	**68**	**124**	**0.58**	**[0.34; 0.97]**	**29.39%**
Heterogeneity: $I^2 = 0\%$ [0%; 90%], $\tau^2 = 0$, $p = 0.520$							
Test for effect in subgroup: $t_2 = -4.52$ ($p = 0.046$)							
**Miscellaneous**							
Ferrando, 2018	93	241	117	244	0.80	[0.65; 0.99]	43.69%
Fernandez Busta, 2020	5	16	4	13	1.02	[0.34; 3.03]	2.39%
**Random effects model**	**98**	**257**	**121**	**257**	**0.81**	**[0.47; 1.39]**	**46.08%**
Heterogeneity: $I^2 = 0\%$, $\tau^2 = 0$, $p = 0.682$							
Test for effect in subgroup: $t_1 = -4.94$ ($p = 0.127$)							
**Random effects model**	**181**	**730**	**263**	**736**	**0.70**	**[0.58; 0.84]**	**100.00%**
**Prediction interval**						**[0.54; 0.91]**	
Heterogeneity: $I^2 = 7\%$ [0%; 61%], $\tau^2 < 0.01$, $p = 0.380$							
Test for overall effect: $t_{11} = -4.16$ ($p = 0.002$)							
Test for subgroup differences: $\chi^2_3 = 8.45$, df = 3 ($p = 0.038$)							

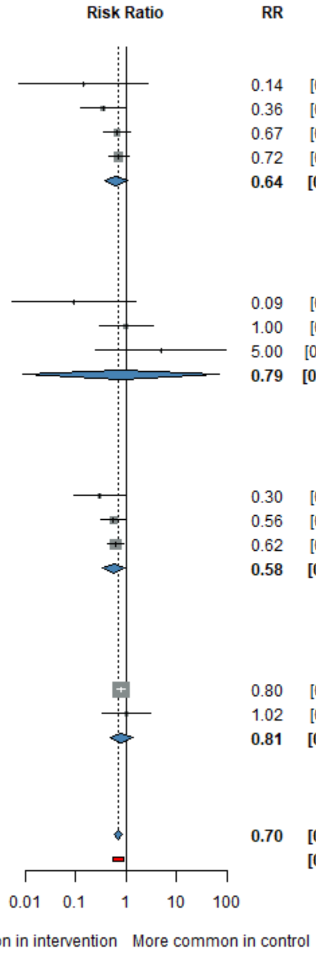

Supplemental Digital Content, Figure S13 – P/F ratio subgroup analysis

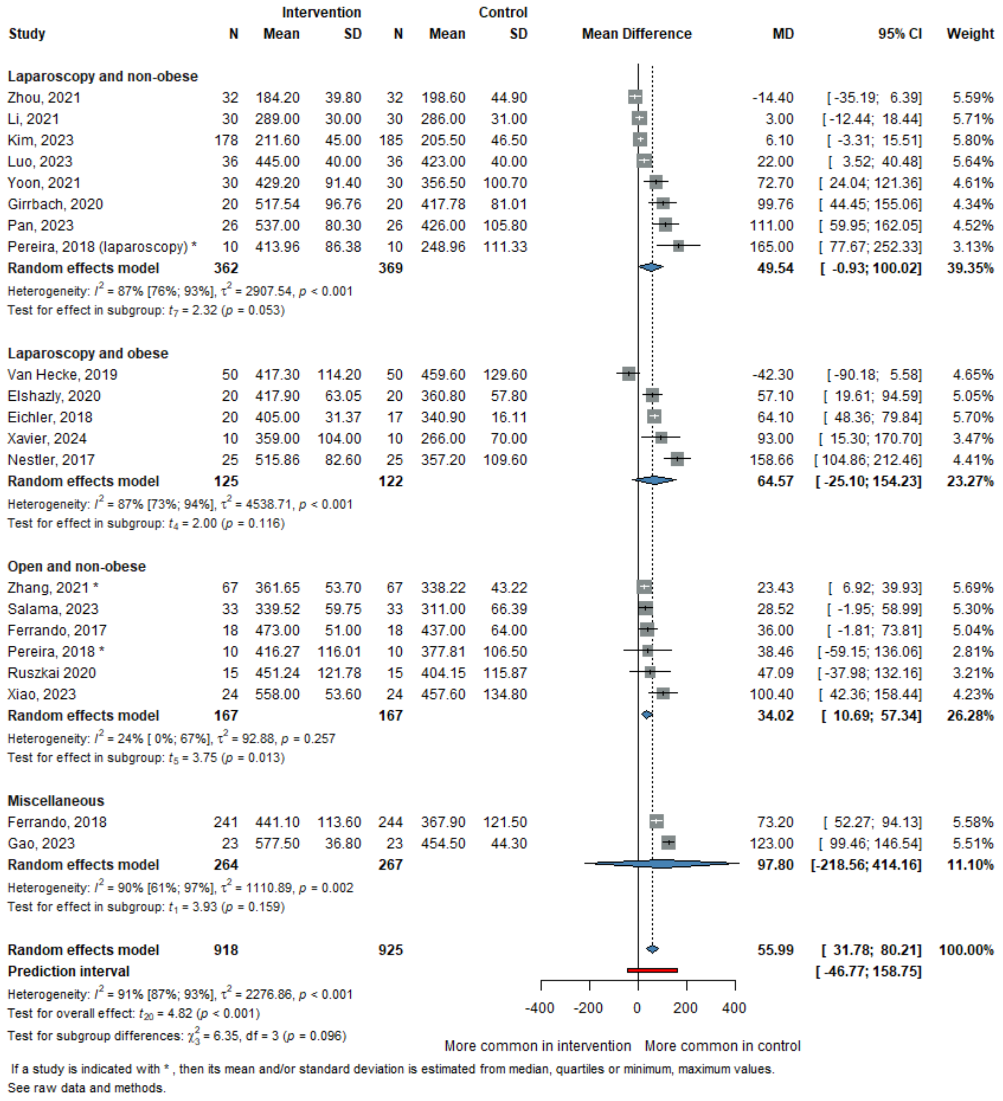

Supplemental Digital Content, Figure S14 – PEEP values, subgroup analysis (cmH2O)

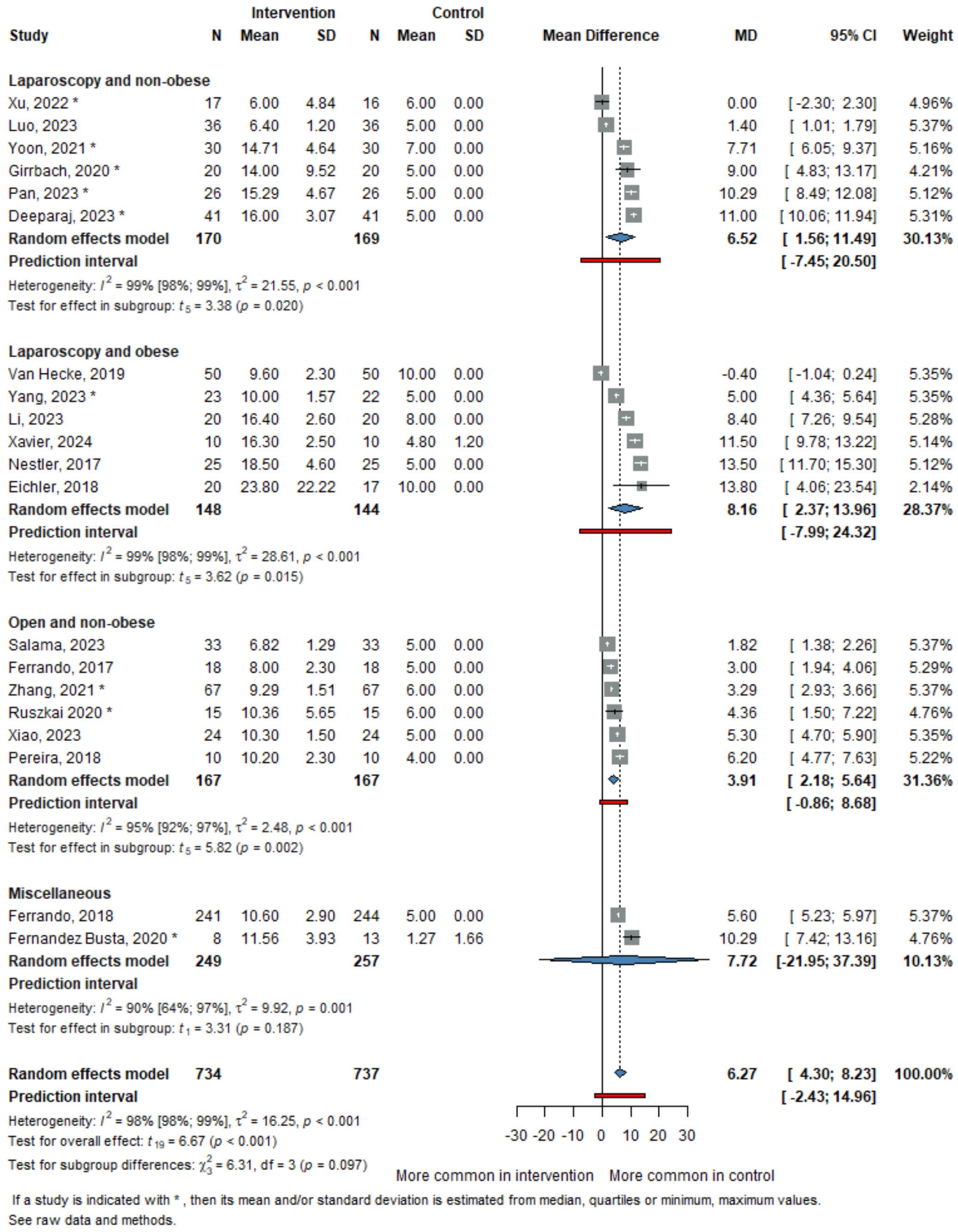

Figure S15 Searching strategy used in the four medical databases on 14 Oct 2021 and 26 Apr 2024.:

Search key for MEDLINE (via Pubmed), Web of Science, and CENTRAL:

(("electric impedance" OR "electrical tomography" OR "electric impedance tomography" OR "electrical impedance tomography" OR impedance or EIT or ultrasound or "ultra sound" or "ultrasound-guided" or "esophageal pressure" or "oesophageal pressure" or "esophageal catheter" or "oesophageal catheter" or "lung ultrasonography" or "lung ultrasound" or "transpulmonary pressure" or "best compliance" or "respiratory compliance" or "lung compliance" or "static compliance" or "PEEP/FIO2" or "dynamic compliance" or "driving pressure" or "Compliance-guided" or "PaO2/FIO2" or "PES-guided" or "individualized" or "individually" or "titrat*") AND (surgery or abdominal or laparasc* or laparosc* or surgical or surgerys or surgeries or pneumoperitoneum) AND (peep or "positive end-expiratory pressure" or "positive end expiratory pressure" or "PEEP-titration")) and random*

Searching strategy in Embase:

('electric impedance'/exp OR 'electric impedance' OR 'electrical tomography' OR 'electric impedance tomography' OR 'electrical impedance tomography'/exp OR 'electrical impedance tomography' OR 'impedance'/exp OR impedance OR eit OR 'ultrasound'/exp OR ultrasound OR 'ultra sound'/exp OR 'ultra sound' OR 'ultrasound-guided' OR 'esophageal pressure'/exp OR 'esophageal pressure' OR 'oesophageal pressure'/exp OR 'oesophageal pressure' OR 'esophageal catheter'/exp OR 'esophageal catheter' OR 'oesophageal catheter'/exp OR 'oesophageal catheter' OR 'lung ultrasonography'/exp OR 'lung ultrasonography' OR 'lung ultrasound'/exp OR 'lung ultrasound' OR 'transpulmonary pressure'/exp OR 'transpulmonary pressure' OR 'best compliance' OR 'respiratory compliance' OR 'lung compliance'/exp OR 'lung compliance' OR 'static compliance'/exp OR 'static compliance' OR 'peep/fio2' OR 'dynamic compliance'/exp OR 'dynamic compliance' OR 'driving pressure'/exp OR 'driving pressure' OR 'compliance-guided' OR 'pao2/fio2' OR 'pes-guided' OR 'individualized' OR 'individually' OR 'titrat*') AND ('surgery'/exp OR surgery OR abdominal OR laparasc* OR laparosc* OR surgical OR surgerys OR surgeries OR 'pneumoperitoneum'/exp OR pneumoperitoneum) AND ('peep'/exp OR peep OR 'positive end-expiratory pressure'/exp OR 'positive end-expiratory pressure' OR 'positive end expiratory pressure'/exp OR 'positive end expiratory pressure' OR 'peep-titration') AND random*

Table S1 – PICO framework

Patients/Population	Inclusion criteria	Exclusion criteria
	Adult patients (≥18 yrs, both sexes) undergoing major gastrointestinal, gynecological, urological surgery under general anesthesia.<br>Both laparoscopic and open; elective and non-elective surgeries, regardless of duration will be accepted.	-Children (<18 yrs)<br>-Patients ventilated for other reasons than abdominal surgery (e.g. ARDS, respiratory failure).
**Intervention**	Mechanical ventilation with individually titrated PEEP setting regardless of the chosen titration method.	
**Comparator**	Conventional (fixed) PEEP setting or zero PEEP (ZEEP) strategy.	
**Outcomes**	Primary outcome:<br>Incidence of postoperative pulmonary complications (PPCs), as reported by the individual studies.<br>1) proportion of patients with PPC<br>2) total number of PPCs occurring in each group<br>3) number of complications included in the definition of PPC (e.g atelectasis, respiratory infection, ARDS, pleural effusion)<br><br>Secondary outcomes:<br>1) Intraoperative oxygenation (PaO_2/FiO_2 ratio; oxygen saturation)<br>2) respiratory mechanics (dynamic and static compliance, driving and plateau pressure),<br>3) vasopressor need (number of patients requiring vasopressor treatment intraoperatively; total amount of vasopressors administered per treatment group)<br>4) duration of surgery (hours)<br>5) postoperative inflammatory response (procalcitonin, C-reactive protein, interleukins)<br>6) length of stay (ICU, hospital)<br>7) overall mortality	

Supplemental Digital Content,Table S2: **GRADE Assessment - Summary of findings**

Individualized PEEP compared to fixed PEEP for patients undergoing abdominal surgery

Patient or population: patients undergoing abdominal surgery

Intervention: individualized PEEP

Comparison: fixed PEEP

Outcomes	Anticipated absolute effects* (95% CI)		Relative effect (95% CI)	№ of participants (studies)	Certainty of the evidence (GRADE)	Comments
	**Risk with fixed PEEP**	**Risk with individualized PEEP**				
Postoperative pulmonary complications (PPCs) follow-up: 7 days	357 per 1000	**250 per 1000** (207 to 300)	**RR 0.70** (0.58 to 0.84)	1466 (12 RCTs)	⊕⊕⊕◯ Moderate[a]	
End of surgery PaO2/FiO2 (P/F) ratio (P/F ratio)	The mean end of surgery PaO2/FiO2 (P/F) ratio was **0** mmHg	MD **55.99 mmHg higher** (31.78 higher to 80.21 higher)	-	1843 (20 RCTs)	⊕⊕⊕◯ Moderate[b]	
PEEP values used (PEEP)	The mean PEEP values used was **0** cmH2O	MD **6.27 cmH2O higher** (4.3 higher to 23.54 higher)	-	1471 (20 RCTs)	⊕⊕◯◯ Low[b]	
Number of patients need vasopressor	548 per 1000	**586 per 1000** (548 to 624)	**RR 1.07** (1.00 to 1.14)	1261 (14 RCTs)	⊕⊕⊕◯ Moderate	
Maximum dose of norepinephrine	The mean maximum dose of norepinephrine was **0** ug/kg/min	MD **0.19 ug/kg/min lower** (2.4 lower to 2.01 higher)	-	157 (4 RCTs)	⊕⊕◯◯ Low	
Lung compliance (dynmic) (Cdyn)	The mean lung compliance (dynmic) was **0** cmH2O	MD **3.26 cmH2O higher** (0.08 lower to 6.61 higher)	-	917 (11 RCTs)	⊕⊕◯◯ Low[b]	
Lung compliance (static) (Cstat)	The mean lung compliance (static) was **0** cmH2O	MD **11.92 cmH2O higher** (6.4 higher to 17.45 higher)	-	656 (11 RCTs)	⊕⊕◯◯ Low[b]	
Driving pressure (Pdriving)	The mean driving pressure was **0** cmH2O	MD **2.75 cmH2O lower** (3.95 lower to 1.55 lower)	-	1530 (15 RCTs)	⊕⊕◯◯ Low[b]	
Plateau pressure (Pplateau)	The mean plateau pressure was **0** cmH2O	MD **2.49 cmH2O higher** (1.08 higher to 3.9 higher)	-	1762 (18 RCTs)	⊕⊕◯◯ Low[b]	

Supplemental Digital Content,Table S2: **GRADE Assessment - Summary of findings**

Individualized PEEP compared to fixed PEEP for patients undergoing abdominal surgery

Patient or population: patients undergoing abdominal surgery

Intervention: individualized PEEP

Comparison: fixed PEEP

Outcomes	Anticipated absolute effects* (95% CI)		Relative effect (95% CI)	№ of participants (studies)	Certainty of the evidence (GRADE)	Comments
	**Risk with fixed PEEP**	**Risk with individualized PEEP**				
Duration of anesthesia (min)	The mean duration of anesthesia (min) was **0** min	MD **0.49 min higher** (6.08 lower to 7.06 higher)	-	1822 (19 RCTs)	⨁⨁⨁⨁ High	
Duration of surgery (min)	The mean duration of surgery (min) was **0** min	MD **6.24 min higher** (2.16 lower to 14.64 higher)	-	1299 (13 RCTs)	⨁⨁⨁◯ Moderate	
Length of hospital stay	The mean length of hospital stay was **0** days	MD **0.06 days higher** (0.71 lower to 0.59 higher)	-	1699 (14 RCTs)	⨁⨁◯◯ Low[a]	
Length of ICU stay	The mean length of ICU stay was **0** days	MD **0.1 days lower** (2.7 lower to 2.51 higher)	-	626 (4 RCTs)	⨁⨁◯◯ Low[a]	
28-day mortality	7 per 1000	**7 per 1000** (3 to 17)	**OR 1.00** (0.41 to 2.46)	850 (4 RCTs)	⨁◯◯◯ Very low[b]	
Total amoung of ephedrine used (mg)	The mean total amoung of ephedrine used (mg) was **0** mg	MD **0.22 mg higher** (1.23 lower to 1.68 higher)	-	323 (6 RCTs)	⨁⨁⨁◯ Moderate[b]	
Total amount of phenilephrine used (mcg)	The mean total amount of phenilephrine used (mcg) was **0** mcg	MD **0 mcg** (0 to 0)	-	416 (6 RCTs)	⨁⨁◯◯ Low[b]	

***The risk in the intervention group** (and its 95% confidence interval) is based on the assumed risk in the comparison group and the **relative effect** of the intervention (and its 95% CI).

CI: confidence interval; **MD:** mean difference; **RR:** risk ratio

GRADE Working Group grades of evidence
High certainty: we are very confident that the true effect lies close to that of the estimate of the effect.
Moderate certainty: we are moderately confident in the effect estimate: the true effect is likely to be close to the estimate of the effect, but there is a possibility that it is substantially different.
Low certainty: our confidence in the effect estimate is limited: the true effect may be substantially different from the estimate of the effect.
Very low certainty: we have very little confidence in the effect estimate: the true effect is likely to be substantially different from the estimate of effect.

Explanations

a. Selection bias may be present

b. Statistical heterogeneity
